# Micropolitics of Mental Health Recovery: An Assemblage Analysis of People’s Experiences of Becoming Well

**DOI:** 10.1007/s10597-024-01311-5

**Published:** 2024-06-26

**Authors:** Jan Georg Friesinger, Alain Topor, Gunnhild Ruud Lindvig, Inger Beate Larsen

**Affiliations:** Department of Psychosocial Health, University of Agder, Grimstad, Norway

**Keywords:** Mental Health, Recovery, Place, Assemblage, Micropolitics

## Abstract

Mental health recovery takes place in a social and material world. However, socio-material contexts have often been absent from recovery studies. The present study was conducted in Norway, a Scandinavian welfare country. We interviewed people at meeting places who had experiences as service users, focusing on their experiences of becoming well, and analyzed their recovery stories using an assemblage framework. Our analysis identified four constitutive dimensions that promote mental health recovery: *an atmosphere of togetherness*, *doings as more than the act*, *personal development*, and *integration in society.* We discuss how these dimensions might be seen as social, relational, and material forces that create important micropolitics that challenge the individualistic professionalization of the recovery concept.

## Background

As in most other high-income countries, a process of de-hospitalization regarding mental healthcare (Fakhoury & Priebe, 2007) was initiated in Norway in the late nineties. The process was officialized in a parliamentary decision to grant 24 billion Norwegian kroner (NOK) for a period of eight years, initiated by the Ministry of Health and Care Services (HOD, 1998). The aim was to develop more community-based ways of helping people with mental health problems. The investment was extended for another two years to reach the goals of the national mental health program, for a total of 30 billion^1^ NOK (Ose et al., 2012).

This investment, by a Scandinavian welfare country, made important changes possible in the institutional landscape for people with severe mental health problems. These changes affected their everyday lives in both material and social environments (Pedersen & Kolstad, 2009). However, studies regarding how and whether these political decisions and the changes they have induced play any role in the recovery processes of individuals remain lacking. Nevertheless, the Norwegian Ministry has begun a new national mental health program with a central focus on health promotion and prevention initiatives and community-based services, granting 3 billion NOK for the next decade (HOD, 2023).

The most commonly used definition of recovery as a “deeply personal, unique process of changing one’s attitudes, values, feelings, goals, skills, and/or roles … within the limitations caused by illness” (Anthony, 1993; Leamy et al., 2011) has been questioned (Karadzhov, 2021; Price-Robertson et al., 2017; Ramon, 2018; Topor et al., 2011). Critics have noted three aspects of this definition of recovery that has dominated the psy-fields. First, recovery has been described as an individual process occurring outside social, relational, and material contexts. Second, recovery has been reduced to an internal cognitive process. Third, it has been presented as a process within the given limits of an illness (Topor et al., 2022).

Such criticisms have led to studies contextualizing recovery processes (Harper & Speed, 2012; Tew et al., 2012). These studies have reflected different perspectives, but their common ground has been an endeavor to locate recovery (and mental distress) in a context outside the person or, more precisely, to locate the person in their context. De Ruysscher et al. (2022) studied recovery in a low-threshold meeting place and identified place-making rituals and inclusive hospitality as significant for service users. Doroud et al. (2018) reviewed the role of place in studies and synthesized a central theme: “Place as a context for doing, being, becoming and belonging” (2018, p. 112). Others have reviewed the role of social support as significant for recovery (Bjørlykhaug et al., 2021), sometimes focusing on the contribution of professionals (Ljungberg et al., 2015). The notion of “doings” seems especially important, as people enter their environments and thus themselves through doing something, acting upon or relating to people and things (Lindvig et al., 2022). “Responsivity,” the participation in social exchanges as an equal partner, has been stressed in doings that support recovery (Skatvedt, 2017).

An improved financial situation may entail adding new places to a person’s enabling landscape in the local community as well as renewed possibilities for social interactions with one’s family and other people in a social network (Davidson et al., 2001; Topor et al., 2018). Research in social science has focused even more strongly on participation and social inclusion than in the listed recovery studies. For example, the concept of “recovering citizenship” emphasizes a mutual linkage between the “5 R’s” (rights, responsibilities, roles, resources, and relationships) that a community or society offers and people’s sense of belonging to it (Rowe & Davidson, 2016). Another concept coined by Tew (2013) is “recovery capital,” which borrows ideas from Bourdieu (1984) and other scholars in terms of resources that people possess (e.g., economic, social, relationship, identity, and personal capital). From Tew’s perspective of recovery capital, mental health difficulties are addressed by situational assessments and the strengthening of people’s resources and potentials. The focus on the protection of civil rights and increased access to different forms of capital points towards an understanding of recovery as a process of widening people’s landscapes, opening new experiences in new places or different experiences in old places. These experiences form the bases for people’s sense of self beyond “patienthood.”

Thus, studies have examined the changing institutional conditions delimiting people’s freedom of movement and the possibilities to create personally designed enabling landscapes, places, and networks of places contributing to well-being and recovery (Duff, 2012; Parr, 2008). The concepts of therapeutic, helpful, and enabling landscapes and places have been introduced to clarify the socio-material aspects of recovery processes (Duff, 2012; Gesler, 1992; Kearns & Milligan, 2020). Place and landscape studies have often stressed the importance of social relationships made possible in the different places within a personal landscape (Larsen et al., 2022; Hope et al., 2023; Sells et al., 2006). The range of possible places for a person is a consequence of macropolitical decisions (welfare policies, de-institutionalization of mental health) and local conditions (housing situation, presence of associative life, application of macropolitical decisions) affecting the recovery journey. Normalization, integration, and participation by service users are among the macropolitical ideas present in the two Norwegian parliament decisions mentioned above (HOD, 1998, 2023). These ideas comply with the recovery philosophy, which highlights the need to create social and material conditions to support just such ideas so that people with mental health challenges can exercise citizenship and remain part of society (Nirje, 1985).

In this article, we have used assemblage analysis to explore recovery stories in which specific dimensions at meeting places have been highlighted as recovery-promoting. Thus, we share the ecological perspective that Rose (2018, 2020) has advocated in examining mental distress and recovery with respect to “mundane experiences.” As a personal, relational, social, and material process, recovery from mental health problems is associated with a person’s situation, which consists of interrelated elements such as bodies, things, ideas, and social formations in terms of socio-material assemblages (Larsen et al., 2022).

We consider the concept of assemblage to be an appropriate framework in which to contextualize recovery processes because it integrates social and material features of people’s lives. In other words, humans are in a relationship with their environment in terms of a powerful and material assemblage whereby relationships between human and non-human elements are understood as blending (Bennett, 2010). DeLanda (2016) emphasizes that assemblages can be characterized by the absence of starting or stopping points with regard to interrelationships, with current relationships changing or new relationships emerging. Thus, assemblages are not static formations but rather can be considered flexible arrangements that clarify the complexity and dynamics of people’s lives (Deleuze & Guattari, 1987); recovery might then occur within specific assemblages affecting people’s bodies and lives (Duff, 2014, 2016, 2023). As such, recovering from mental health problems might be understood as a recovery journey through different socio-material contexts (Larsen et al., 2022; Leamy et al., 2011; Topor et al., 2011). Including both human and non-human perspectives in recovery experiences could contribute to the development of a different sense of self.

After reviewing the existing literature on recovery, it becomes apparent that a knowledge gap still exists in the field of recovery studies, particularly regarding the social, relational, and material aspects of the process of becoming well. In our study, we aim to bridge this gap by adopting an assemblage framework and addressing the following research question: How can individuals’ recovery narratives shed light on socio-material relationships that promote the recovery process?

## Methods

The present study is part of a larger research project exploring factors that may promote or hinder recovery from mental health problems, with or without co-occurring substance use, related to both mental health and addiction services and to people’s living situations in general. More details regarding the project can be found in the report by Larsen et al. (2022). In this study, we focused on recovery-promoting experiences. We asked people with experience as users of mental health or addiction services about their own recovery and were interested in their personal experiences related to both the services and their living situation in general. Our aim was to obtain detailed descriptions of the experiences they related; therefore, qualitative methods were well suited. Using an assemblage framework informed by DeLanda’s (2016) realism, we analyzed the participants’ recovery narratives and noted how they emphasized different places and people as significant for them to grasp their lives and the processes of becoming well. Thus, this study has provided knowledge regarding how the participants themselves reported their various experiences of recovery.

### Data Collection

We conducted focus group, paired, and individual interviews to collect data. More specifically, we planned to begin with focus group interviews to explore the topic by allowing the participants to reflect on one another’s narrations and then retain the possibility to interview people from the groups individually regarding topics on which we desired to elaborate. The focus group method centers on dialog among participants, meaning that the interviewer is more focused on listening to the stories the participants tell to one another rather than asking them questions. However, beginning with focus group interviews produced a different result because some participants had agreed to join a group while others had elected to be interviewed in pairs (with a peer) or only individually. In such cases, we considered that the various ways to access the participants’ experience-based knowledge might serve to fill each other’s gaps and thus strengthen the variance and depth of the narratives. The interviews were conducted at meeting places and the university and lasted between 60 and 90 min. They were audio-recorded and transcribed word-for-word by researchers affiliated with the research project, and the total text length was 174 pages (Times New Roman, 12-point type).

### The Participants

We recruited 29 participants from different low-threshold meeting places for people with mental health or addiction problems in different Norwegian municipalities. To begin, we requested the managers of the meeting places to distribute written information regarding the study. We invited participants who considered themselves to be recovered, not necessarily in the sense of clinical recovery but in the sense of having a better life. In total, we recruited 16 female and 13 male participants (Table 1).

**Table 1:** The interview methods and participants (gender)

Interview method	Male	Female
Focus group interview 1	3	4
Focus group interview 2	1	4
Focus group interview 3	2	3
Focus group interview 4	3	3
Paired interview 1	2	
Paired interview 2		2
Individual interview 1	1	
Individual interview 2	1	
**Total**	13	16

Note. Adapted from (Larsen et al., 2022)

### The Interviewers

Altogether, five researchers by profession and one co-researcher with lived experience (an expert by experience) contributed to data production. All focus group interviews were conducted by the co-researcher together with one of the researchers by profession (alternating). The co-researcher led the discussions while the researcher by profession functioned as a moderator. The two interviews of paired subjects were conducted by different researchers by profession, and one researcher by profession conducted the two individual interviews.

### Analysis

Analysis of the collected data was based on assemblage analysis and was conducted in three steps (Feely, 2020; Fox & Alldred, 2015). First, we aimed to identify socio-material relationships and events within the participants’ stories across traditional dualistic thinking, incorporating both humans and non-humans. Second, we mapped parts of the outlined socio-material relationships in the context of mental health recovery. As previously mentioned, the participants highlighted meeting places as significant; as a result, we focused further analysis on them. Third, we explored in further depth the specific dimensions that had enabled recovery and attempted to group them. Despite our focus on recovery promotion, the interviewees mentioned many aspects that had hindered recovery as well, including narratives about being discredited and not believed.

During the analysis, all authors met several times, reflecting on methodological and ethical issues such as respecting the participants’ voices and discussing the appropriateness of our interpretations. We checked the consistency of our findings further by reviewing the analytical steps. The assemblage analysis resulted in the identification of four constitutive dimensions of mental health recovery: an atmosphere of togetherness, doings as more than the act, personal development, and integration in society.

### Methodological Considerations

Malterud (2001) proposed relevance, validity and reflexivity as criteria for qualitative research. In our review of the existing literature, we identified a research gap and highlighted the relevance for our study as it focuses on the neglected socio-material aspects of recovery. In respect to validity, we have illustrated the participants’ experiences through their own narratives of specific cases relating to features that they considered recovery-promoting. Regarding the focus groups, participants’ personal descriptions were narrated in a group; a potential strength of using four focus groups is that the participants mutually supported one another in telling their experiences. This may have been the case for the paired interviews as well. In the two individual interviews, the participants met the same researcher, which was important to them because no sensitive information about themselves was shared with others. We believe that these different methods complemented one another because data provided by participants discussing their recovery experiences is not always identical to what they might relate in one-on-one settings. Thus, by employing a mix of data collection methods, we might capture a wide range of recovery stories from different angles, thereby accounting for the diverse experiences of the participants. To ensure anonymity, pseudonyms were assigned to the quoted participants.

During the project, the researchers remained focused on the service user perspective. For instance, we noticed that the participants became more confident after the introduction of the researcher through experience in the focus groups. We acknowledge that a study focusing on users’ experiences under different conditions with different researchers might meet criticism for a lack of clear structures or for fluctuating information stated by the participants. Nonetheless, we would claim that our methodological choices can rather be seen as ways to account for the varying depth of participant experiences. An additional limitation to consider is the recruitment of individuals who self-identify as being in recovery, because valuable insights could also be obtained from those who do not perceive themselves as such. Regarding reflexivity, our author meetings facilitated discussions on how our diverse professional backgrounds might impact the research process. These discussions helped us in grappling with any preconceived notions of recovery and ensuring a more balanced approach.

### Findings

We explored individuals’ recovery experiences in connection with socio-material relationships at meeting places. The four assemblage dimensions we identified were formed by specific socio-material relationships affecting people’s bodies (and minds). Recovery thereby occurs as forces related to the environment emerge in material and powerful assemblages and seem to establish processes. In other words, the four dimensions describe people in the context of a long and winding journey of mental health recovery. These dimensions are interrelated, and they draw on familiar socio-material relationships; however, each dimension reflects its own emphasis and contribution to the recovery process.

### Atmosphere of Togetherness

When the participants mentioned places where they felt comfortable and safe, they referred to a type of atmosphere. They did not use the word “atmosphere,” but they explained emotions connected to the meeting place itself and the other people there, which were important to them. We view these narratives as descriptions of human and non-human togetherness, in which the participants continuously connected their positive experiences to the places. Frequently, the word *place* was used. Expressions such as “I feel at home,” “We are a family,” and “I feel welcome” illustrate that they felt close to each other in a material world. Olivia described the atmosphere of the place:

I feel at home because, in essence, I know that if I have a bad day, somebody will always come and give me a hug. We support each other. Come on! We work together, and I can’t think of anything negative about the place.

Evidently, recognition of one another as complex people is related not only to kindness but also to equality. The service users and the staff supported each other and cared for each other. Their relationship thereby had a non-hierarchical character, as reported by Lucas, who had experienced being judged because of his problems. Now, he felt included and safe:

Here we are equal, and I love it here. It is a totally different place, and people are honest. People say, “Hi.” It’s a small place [community], and people know each other. It’s great to live here and have this meeting place … It’s good to have this place for those of us who struggle a bit.

When asked about the differences between this low-threshold meeting place and a more specialized place within mental health or addiction services, such as a rehabilitation center, a ward, or an office, Noah explained the following:

Here, you get treatment and help in a different way. When you attend an appointment at a professional place, you know where to sit at the table. These are my experiences. Looking at these professional meetings, nobody gives you a pat on the shoulder. Most people who are visiting this place do not recognize who is employed or not. This feeling, I believe, is the big difference between those places … It is about equal status.

Another man appreciated both the community to which he had moved and the meeting place he frequented. In a way, the positive places that the participants have described are places where they can simply be together as fellow human beings, meaning that they are together in the ups and downs that life offers. The homelike living rooms create atmospheres of togetherness because the interior reflects equality. Emma expressed this idea:

She [the professional] helps me a lot. We are kind of friends; get a hug. Get a coffee. Regardless of my mood, I can say I have a shitty day or a great day, and it is fine … We take care of the place, and we have a place to go to. That means everything. Because if we had been at home all day, we would have focused on the pain instead of sitting here talking.

Within this outlined dimension, the atmosphere of togetherness is a result of relations between human and non-human dimensions, as all meeting places might be understood as assemblages of chairs, tables, a coffee machine, the smell of coffee, the smell of freshly baked waffles and cakes, cups, and the people that have staged the recovery atmosphere of togetherness. Thus, tiny gestures are rendered possible and significant for signaling (mutual) equality and kindness.

### Doings as More than the Act

The participants emphasized the importance of doings, which are always connected to more than just the act. Some participants referred to cooking. Olivia and Kasper said, “You should come Friday, and you will get chocolate fondant.” Another remembered the delicious meal with cabbage he was served by another service user, and one place contained a pleasant kitchen where they cooked and a dining room to serve meals. In addition, the meals were inspired by the different communities or countries from which the participants came. Some sold coffee in the cafés to people in the neighborhood, and others were busy cleaning or chatting.

Overall, the activities in different rooms were interwoven and connected to local communities or other countries. No specific activity was designated as significant, but doing something in terms of being responsible for something, someone, or the place was considered important, as Ella expressed:

Sitting there, sorting clothes [for the second-hand shop], tearing [fabrics] to pieces for rugs. I walk around [in the shop] and make sure everything is well presented.

For some, being involved in the meeting place was a stepping-stone to paid work. For most of them, the doings reflected what they did during the day, but they also reflected what others did to them, as Leah explained:

They [the staff] constantly encourage me. They encourage me to get a driver’s license and stuff like that, so now I am about to get the license. They are nagging me in a positive way; they care … And they pushed me; he called up different places and gave me the phone. And I got a job as a life assistant. Within a week, I became a life assistant.

Having a place to stay and tasks to be performed helped the participants to structure their days. Being (increasingly) responsible for the tasks they assumed strengthened their autonomy. Many participants mentioned a life in which their daily routines had been heavily disrupted prior to their engagement with the (meeting) places. They had slept through the day and watched television at night. Olivia said, “It is not easy to start working at nine o’clock in the morning, but I am mega-happy that I do.” Filling the days with meaningful activities as described at the places was important. Sofie used the following words:

Being allowed to do something sets your mind in place. That you are not just going around and thinking about getting high. Before, I was not willing to stop using drugs. I must be honest: I just stepped into it, and then it was okay. But it’s not okay, when you’re getting high until it’s killing you. Therefore, it’s good to have a place to go.

Moreover, the participants expressed a sense of being trusted to address any tasks by themselves such as Leah summarized:

Together, we run our own place. They trust us, and each of us gets our own key to the workplace. We can do what we want to do … It’s a vote of confidence.

The above case implies an assemblage of recovery, literally exemplified by *keys of trust*. The example also shows the interrelatedness of the promoting dimensions because actions have implications for oneself, for relationships, for the place, and for others. An assemblage of doings is an activity that finds place in different rooms and buildings in which individuals can use material tools and cooperate with others.

### Personal Development

Participants expressed that building a sense of self is a significant part of recovery and might be experienced as a consequence of both human and non-human forces. Human forces were the people in the meeting places and in society at large, and non-human forces might be pleasant interiors or surroundings that signaled love and respect, as well as places in the centers of towns and not at their margins. Expressions such as “nobody dislikes being trusted” illustrate meeting places that give people the possibility to grow and that have not been narrowed by any stereotypes linked to mental illness or drug addiction. During a focus group interview, the participants discussed the importance of the work on the complicated path of recovery, which must be performed by themselves. Leah stated the following:

Before starting here, I did not have many expectations. What should I say, then? I hope to become somebody, and I hope that we have a nice time. However, we have it more than nice here. I do not know what I would have done without the place now … The staff, for instance, supported me to do things I hadn’t done for years … I smoked a lot before but have quit now.

Nevertheless, to grow as a complex person and build a sense of self beyond the diagnosis entails more than simply filling days with activities. Olivia expressed this idea:

It is nicer to be here together and give something to society. To show with a “Hello” that we matter is pleasing. How should you respond? People have called us shitty drugheads and worthless. But here we come!

Her statement highlights the fact that having a physical place to stay, people to be together with, and something to do as a group had supported her personal development. Moreover, hope for a positive future was an important subject that was encouraged in these places. Hope was linked with concrete possibilities, mediated through the arrangement of the different places, to “create something,” “become somebody,” “avoid worrying about being worthless,” or “achieve feelings that affect you in a good way.” When asked whether participating in the meetings had improved other parts of life as well, not all participants could answer affirmatively. However, some clearly described changes in life. For example, Ella explained that people stated that she had become more open since she had begun attending the meetings:

I feel I have grown, and this gives me a lot. I got help from a mental health professional [nurse] over more than a decade, and I realized that I had not come so far. Therefore, I swapped my mental health professional, and suddenly I understood my challenges. It was confirmed that I have developed and changed from before, and that is because of this place here.

Despite stories of mental health or addiction problems, hope was linked to the actions made possible in the shops or cafés, which had also motivated and helped some participants to believe in themselves and apply for jobs. Using his own example, Isak explained how he earlier had thought that he did not fit in the labor market: “I am not able to work and cannot get a regular job. But I have stopped thinking that way.” This strengthening of self-confidence was confirmed by other participants, because many had hardly believed they could get an ordinary job, often not because of themselves but because of a system that did not desire a curriculum vitae with gaps. Over time, within places with recovery-promoting assemblages, the participants had regained hope and had developed a sense of self that had made them feel like people rather than “shitty drugheads.” In the words of Jakob, “What on earth … Why should I not be able to, right? The impact there that I experienced was that I can do more than I believe.” Personal growth was described in relations between human and non-human dimensions.

### Integration in Society

As the meeting places were located in and open to the larger society, the participants could change their identity as outsiders and become insiders among people in general. Insider positions promote personal development.

In addition to experiencing an atmosphere of togetherness, focusing on doings more than the act, and developing personally, the participants described feeling integrated in the local community. They expressed that, over time, they had developed a sense of (socio-material) belonging not only with the people and things inside the meeting places but also with the surroundings and neighborhood. For example, Ella who had been sorting clothes was connected to the local community because people in the neighborhood were giving items to the second-hand shop or buying items there.

At one meeting place, this openness towards the local community was produced through the offering of free rental services for different types of sports and outdoor equipment. These rental services were offered to all people from the local community who could not buy or did not wish to buy their own sports and outdoor equipment or wanted to test equipment before buying their own. Leah stated the following:

The idea is to take care of the environment, and we all might need such equipment but not necessarily need to own it.

The free rental service had been designed particularly for people with low economic resources, but with its open invitation for all citizens, it also led to the creation of mutual ties between the meeting place and the community. This openness reported by the participants can be seen as a support for increasing participation and inclusion in wider society.

In a more nuanced way, the meeting places that the participants described were interconnected and assembled as a landscape ranging from places “where you can be yourself” to places “where I have to cope with my pain alone,” as Olivia said. However, it was also a place for reciprocal interactions beyond diagnosis and shortcomings. Leah reflected as follows:

I have started to feel more at home here than I did in the beginning; however, I cannot say that I have found my place. But, at least I have found one place.

Concerning interconnectedness, Sofie who loved to be social but faced challenges in going out explained that she had finally been able to visit a mall with a co-worker from the meeting place. In the words of Jakob, “The [social] meaning of the place goes beyond it, and it is spread over to the outside.”

Moreover, participants mentioned that, on the one hand, they had met new people at the places and had established social relationships with them. On the other hand, they had informed their friends and relatives about the places, and some of them had begun to visit and even work there. Viewed through the assemblage lens, the latter condition can be understood as a form of socio-material embeddedness, in which a meeting place is always situated in a community and the built or natural environment. For instance, Noah described some low-threshold exercises at a gym inside a meeting place at which running was performed in the local neighborhood and parks. For him, running was more than a bodily activity because it created, through mobility, a promoting space that connected the inside and outside of the meeting place together. The goal of the training was to participate in an annual running event with others, which can be viewed as community involvement that had begun at the meeting place.

## Discussion

As outlined above, a recovery assemblage can be represented as an atmosphere of togetherness, doings as more than activity, personal development, and integration in society. These findings may be considered in the light of macropolitics that have inspired society to downsize asylums and move people with mental health challenges out of hospitals and into municipalities for more community-based care. The political system has highlighted this process as normalization and integration (HOD, 1998). Our findings can also be understood as micropolitical expressions of how this idea of downsizing has come into action at meeting places in Norwegian municipalities. In this section, we discuss how the assemblage dimensions mentioned above might be seen as forces that create important micropolitical effects with implications for mental health services and the potential to challenge the professionalization of the recovery concept.

The term “micropolitics” refers to a domain of activities that have an impact on people’s lives and are usually taken for granted (Bennett, 2004). Having people nearby and somewhere to stay (the socio-material) is a matter of course for most people, and they may never consider how important or useful these elements are. Micro- and macropolitics are linked to each other, with the difference being that the functions of the latter belong to a governmental level whereas micropolitics relates to local levels, such as municipalities, neighborhoods, and services such as meeting places. According to new materialist perspectives (Bennett, 2010; DeLanda, 2016; Duff, 2014; Fox & Alldred, 2015), socio-material relationships in assemblages possess “capacities to affect and be affected.” The very presence of the people we interviewed in the community and at the meeting places was the result of macropolitical decisions mediated at a micropolitical level (Deleuze & Guattari, 1987).

In this study, we observed micropolitics that had affected participants’ internal and external lives and their material surroundings. The participants at the meeting places all mentioned their personal development that had occurred in connection with being present at these particular places. The meaning of having something to do (doings as more than the act) together with other people they trusted (atmosphere of togetherness) was highlighted as a dimension of a recovery assemblage. The equal status between those who were mentioned as having mental health or addiction issues and those who did not have these labels was appreciated. Other dimensions they described were the importance of feeling at home at the meeting place itself and the way in which the meeting places were open to society (to be affected), thus offering possibilities to widen one’s enabling landscape further with new places beyond those created to help people with mental health and addiction problems (Hope et al., 2023). In Tew’s (2013) perspective, one could state that such recovery-promoting dimensions can be interpreted as a mobilization of forms of recovery capital. One of the participants used the meeting places as a stepping-stone to paid work; others preferred to continue working for free at the meeting places. Hence, for some, to be integrated in society meant to be integrated in the meeting place itself; others had also become a part of the larger society. What is certain is that when the participants discussed recovery, they linked their stories to the social, relational, and material contexts of everyday life (Topor et al., 2022) rather than to what had occurred in a therapist’s office. This might challenge professionalization.

### Micropolitics of Recovery Challenge Professionalization

One might inquire how these outlined micropolitics of mental health recovery reflect a shift in the understanding of recovery. In our opinion, applying the assemblage lens to (mental) health is useful because it acknowledges ways of becoming well in different socio-material contexts. By contrast, research literature regarding mental health recovery still relies heavily on Anthony’s (1993) individualistic recovery notion and clinical understandings that were implemented in mental health services in recent decades. The implementation of such recovery positions can be seen as professionalization of the recovery concept (De Ruysscher et al., 2019) and colonization of users’ voices by only incorporating successful recovery stories (Llewellyn-Beardsley et al., 2022). Thus, recovery research could be criticized for its widespread use of the perspectives of professionals (Rose, 2014; Topor et al., 2022).

In other words, a micropolitical understanding of mental health recovery confronts professionalization with the notion of socio-material relatedness and the non-linearity of recovery stories across interactive and conflicting forces. A portion of the recovery literature has been unable to deliver a critical variation of knowledge to examine mental health (issues) and its services in depth. For example, in a meta-synthesis of the role of place in recovery processes by Doroud et al. (2018), professionals have narrowed the definition of places according to their occupational understanding and detached from the societal and micropolitical forces that are essential in the making of recovery-enabling places. As reported by participants in this study, to grow as a person and build a sense of self beyond the diagnosis is not an easy journey without struggles and turbulence but could also be seen as a journey in which “a thousand tiny benefits” take place.

The micropolitical aspects of mental health recovery include both non-healing and healing issues; as the study participants expressed, the fundamental difference between meeting places in the community and professional mental health services is underpinned by colonized knowledge, in which user experiences have been displaced (Rose, 2018). Thus, the circumstances under which a person is recognized as equal do matter, as highlighted by the citizenship approach of Rowe (2015). As shown in our findings, meeting places organized non-hierarchically (such that one can hardly realize who is employed or not) or organized to blur the traditional borderline between health or normality and illness or deviance were outlined as helpful by service users. This observation goes beyond the usual dyadic relationship between service user and healthcare professional and beyond services in which the system and its employees seem more important than the people for whom the services are intended. The degree to which power issues have lately become important for the understanding of mental distress can be seen in the attempts to challenge the diagnostic system with the Power Threat Meaning Framework (Johnstone & Boyle, 2018; Pilgrim, 2020; Read & Harper, 2020), which does not consider people’s responses to threats as symptoms.

In this regard, we propose continuation of the critical examination of mental distress and recovery through assemblage lenses with micropolitical sensitivity (Fox & Powell, 2021a, b) to develop knowledge that might help decolonize the professionalized system by revealing its diagnostic foundation and taken-for-granted power structures, including those places that represent the system. Using assemblage concepts, mental health recovery can overcome models underpinned by knowledge of psychiatric rehabilitation (Dell et al., 2021). Instead of continuing to colonize people’s experiences of mental distress and thus supporting politics of knowledge that creates underclasses (Wacquant, 2022), why should one not assemble more enabling places (Duff, 2012) and landscapes in line with what our participants have described? As indicated above in the introduction to the recovery literature, the answer is ambiguous.

We would argue that places with promoting micropolitics impact democratic society as well, including diversity and well-being and working as counterparts to territorial stigmatization (Slater, 2021). Such locations include affordable cafés, shops, or meeting places, as shown by our findings and those of others (Duff, 2016; Parr, 2008); areas with improved economic conditions; safe homes in neighborhoods with amenities (Friesinger et al., 2019, 2022; Lupton & Lewis, 2022); green areas with possibilities for animal companions; bike services (Marks, 2021); and others. All these places with different qualities point towards the community and can be understood broadly as enabling landscapes that de-territorialize and signal a community of people. Future mental health programs therefore should address such issues and allow sufficient financial support for community approaches, which is not the case for Norway’s newest national program, which only accommodates 10% of previous applicants (HOD, 1998, 2023).

Meeting places that involve strong user participation and openness in terms of accessibility by the local community (with low running costs and low thresholds) have particular potential to decolonize mental health services. This socio-material relatedness that enables the community at such places helps to develop promotional ties. Nonetheless, places promoting the micropolitics of mental health recovery are also under pressure as niches (Bister et al., 2016) because of their (symbolic) power to bring societal change; they might therefore be considered as opponents to ideas based on the politics of neoliberalism, classifications, and psychiatrization (Rose, 2020; Slater, 2021; Topor et al., 2022; Wacquant, 2022; Wacquant et al., 2014).

Further research is needed to explore the interconnected facets that support mental health recovery, including the implementation of emancipatory politics that address the range of individuals’ recovery experiences and its socio-material foundation. While our study has certain methodological limitations, such as the recruitment of individuals who self-identify as being in recovery, the dimensions found to promote recovery can potentially be applied to comparable contexts in other communities.

## Conclusions

In this article, we have examined, within an assemblage framework, stories of socio-material relationships to (meeting) places that affect people’s mental health recovery. We have shown that mental health recovery not only is relational and situated but also is affected by conflicting and interacting forces that together construct micropolitics that promote individual journeys to becoming well. These micropolitical elements of mental health recovery can further be described through four interconnected dimensions: *an atmosphere of togetherness, doings as more than an activity, personal development*, and *integration in society*. These constitutive dimensions are intended not to be the final state of the art but rather to act as an invitation for further critical research regarding this subject. Through assemblage thinking, simplistic underpinnings of mental health recovery can be challenged and power issues in situations can be highlighted.

## Footnotes

1 Around 2.8 billion dollar (currency rate, June 23).
